# Mr. Ring, on Vaccination

**Published:** 1806-10

**Authors:** John Ring

**Affiliations:** New Street, Hanover Square


### *To the Editors of the Medical and Physical Journal.*

Gentlemen,

PERMIT me to add my congratulations to those of Dr. Adams, in your last Number, on the great improvement which has lately taken place in your Journal, with regard to politeness of style, and at the same time to express a hope, that another improvement may hereafter take place, that gentlemen will write on subjects which they understand. If, however, any one should again come forward, and expose his ignorance, and be vanquished by a more able antagonist, instead of appearing crest-fallen, and acknowledging his defeat, he may brandish his quill with an air of triumph, and exclaim, "Think not, gentle Reader, that I am sensible of any inferiority in this contest; my opponent is more than a match for me in the 'Vocabulary of Abuse,' and as I am *well bred*, I shall retire."

This excuse, old and stale as it is, may still serve, for want of a better; and the most hardened sinner in the republic of letters may plead it without a blush. The most sarcastic writers are not always the most patient under the lash; and a scribbler has been known to declaim against his adversary's talent for abuse, who has the whole vocabulary of abuse at his fingers' ends.

But it is time to dismiss this subject. I now beg leave to adduce further evidence in favour of Dr. Jenner's opinion concerning the origin of the cow-pox, in opposition to that of Dr. Kinglake, who supposes that it originates from the small-pox. In addition to arguments already brought forward to the contrary, one more, and that of no small weight, may be drawn from Mr. Dalton's letter, which I promised in your last Number to bring forward.

Mr. Dalton tells us, that as far as he can learn, horses in India are not liable to the grease; and, consequently, there is no opportunity of trying its effects on the cow, or the human species, with recent equine matter. He also informs us, that he has not been able, by a minute and particular search, to discover the cow-pox in the cow.—Hence, the preservation of cow-pock matter, in that country, by a regular succession of patients, is an object of the greater importance. He remarks, however, that the governments in India have given vaccination a liberal support, in order to diffuse the practice; so that we have reason to hope they will preserve this vestal flame, so pregnant with advantage to the human race. He

He informs us, that about half a million of the natives had then received the benefit of vaccination: and at least three thousand of them had afterwards been put to the test of variolous inoculation. He himself had vaccinated about 15,000 patients, and put many of them to the variolous test; but had only been able to produce a slight local inflammation, and that only in a few of them.

To return from this digression, which, I trust, will be excused, on account of the interesting information which it affords, I shall now state the purport of Mr. Dalton's letter in the Madras Gazette. From this it appears, that the author of the letter in the Madras Courier had expressed a conjecture, similar to that so often expressed in this and other parts of the world, and one which seems the most plausible, if reasoning a priori be admitted as proof, instead of facts; namely, that the cow-pox proceeds from the small-pox, variolous matter being applied to the teats of the cows by the hands of the milkers.

In one respect, however, this writer differs in opinion from others who have advanced this hypothesis; for he thinks it probable that the mildness of the cow-pox may be owing to the mildness of the small-pox in the milker, from whom infection was received; whereas others think it is rendered mild by transmission through the juices of the cow; an opinion which does not appear to be warranted by observation.

As a further confirmation of this position, Mr. Dalton published in the Gazette, the result of repeated experiments made by him in the government gardens at Madras in 1802, by desire of Earl Powis; in order to ascertain whether it was practicable to produce the cow-pox by means of variolous matter inserted into the teats of cows with a lancet, or absorbed in consequence of friction.

To render these experiments as complete and satisfactory as possible, several milch cows were selected; and some of them, in the presence of his lordship, were inoculated by Dalton, in their teats and udders, with the most active variolous matter that could be procured; while the teats of others were rubbed with it for a considerable time, till they became highly inflamed.

No pustule was excited on any one of the cows inoculated; but ulcerations appeared on the teats, into which matter was rubbed, the third day after the frictien. Several young children were inoculated with the matter; and their arms inflamed and festered. They had also a slight degree of fever; which gave Mr. Dalton hopes that his experiment experiment had succeeded, and that he had produced a mild species of small-pox; but on putting them to the test of inoculation, they all had it in the most indubitable manner, and regularly went through the disease. Mr. Dalton concludes his paper with observing, that all the circumstances which he has stated will bear the strictest scrutiny, as they are well known to several medical practitioners at Madras.

I now beg leave to offer a few remarks, in reply to those of Dr. Walker, in your Journal for June; who, before he attempts to vindicate the opinions advanced by himself, thinks it necessary to censure those advanced by others. He says, there is a danger of an extremely serious nature, attending the practice of vaccination, which he is afraid I, and other labourers in the vineyard, treat too lightly,—that of exciting an alarm in the minds of parents, and destroying that confidence which Dr. Walker thinks might so easily be afforded them, concerning the future lot of their children.

This is, indeed, a serious charge; and if any man can convict us of it, he will render an essential service to the community at large. It is, however, worthy of notice, that while we are accused by a fellow labourer of inculcating too much caution, we are accused by our opponents of a different line of conduct, that of prosecuting our object without any caution at all. These charges are contradictory to each other, and shew the difficulty of pleasing all the world. How far they are well founded, others must determine.

Dr. Walker advises me, not to encumber myself with all the instructions of all the vaccine societies, as well as individuals, and excite or increase doubts, by declaring what is not a test of security; but to declare what is a certain test, and to propose a more simple one than the induration about the tenth day. Dr. Clarke, of Nottingham, has anticipated the remark, which is so obvious on this occasion, that we are not precluded from pointing out the fallacy of one test, merely because we may not be able to point out a better. But if I were under a necesity of pointing out a criterion, it should not be the induration as proposed by Dr. Walker, nor the areola as proposed by others; but the peculiar scab, as described in the instructions of the Jennerian Society, and in other publications on the same subject.

This criterion of the true disease, even if others be allowed to be equally certain, has one advantage; which is, that that it never can exist where any considerable violence is offered to the vesicle; an occurrence which, I am sorry to say, is still too frequent, and that even from the rude hand of the inoculator cutting up the pustule, scooping out its contents, and thus interrupting the regular course of this innocent process.

That the induration is no certain proof of security, as Dr. Walker supposed, is evident from Dr. Clarke's Report in your Journal for August; by which it appears, that it failed in three cases out of thirty-five. I am therefore happy to find, that Dr. Clarke, and the other gentlemen of the Vaccine Institution at Nottingham, agree with me in opinion; and are determined to have recourse to a re-inoculation with cow-pock matter, in all doubtful cases. That the cases considered as certain by Dr. Walker are in reality uncertain, needs no further proof.

Dr. Walker sets but little value on the test of inoculation with vaccine virus; and tells us, on the authority of a surgeon from Buckingham, that in one case it did not succeed till the twenty-ninth time. This, however, must have been the fault of the inoculator, or of the matter; for it is now very well known, that when the matter is good, and the operation properly performed, it seldom fails. I could, indeed, produce two instances, if necessary, in which the patients had been unsuccessfully vaccinated eight times; the first by two practitioners, and the last by one; yet I successfully vaccinated them both, in both arms, the first time. This renders it probable, that the three practitioners who had inoculated them before, though otherwise respectable, were not conversant with the practice.

Dr. Walker says, every one who is much acquainted with vaccination, must have observed *a considerable quantity of pus* is often produced in the centre of the pock by the wound of the lancet. Hence he thinks it necessary to pursue certain means, such as removing the scab, and wiping out the pus. I am neither convinced of the frequent occurrence of any such circumstance, nor of its occurring at all, unless vaccination is performed in a rude and unskilful manner, or the vesicle injured in an early state. Even when it occurs, I know by experience that it is not necessary to adopt the practice which Dr. Walker recommends. The smallest particle of lymph, if properly inserted, is capable of producing infection; and I venture to affirm, that there are but few cases of genuine cow-pock, where this cannot be obtained.

With

With respect to the thousands, who, as Dr. Walker tells us, have had their pocks on each arm broken down from day to day, at the Central House, and, as far as possible, completely exhausted, for the sake of supplying the demand for matter, I am happy to find that the shield of Jenner has, in general, proved their protection. But let us not put the strength of that shield to an unnecessary or too severe a trial. The Nottingham cases, and many others on record, shew that the vesicle cannot at all times be totally deranged, and exhausted, with safety.

As to the task of supplying the demand for matter, there is no difficulty in this which may not be surmounted, by the means already proposed in this Journal, and other publications.

Dr. Walker informs us, that if he were about to take matter from such a pock, as that exhibited in my Treatise, he should think it right to remove the scab, and wipe out the little subjacent drop of pus, in order to guard against impurity. Here, I can declare with confidence, that his fear is imaginary; and that it is Dr. Walker himself, who creates a vain alarm. Having occasionally supplied Dr. Jenner, and almost all the principal inoculators in this kingdom, with vaccine matter in a fluid and a dry state, sometimes in the arm, and sometimes on other vehicles, and having also furnished a large quantity to the army, and to practitioners in other parts of the world, I can with truth declare, that I do not recollect a single instance, in which I have ever heard any complaint of its being mixed with pus, or producing a spurious pustule.

Had there been any grounds for Dr. Walker's apprehension, such occurrences must have been frequent. I therefore conclude, that where the vesicle is in any degree converted into a pustule, it is generally, if not always, owing to some injury done to it at some preceding period. As to the idea of Dr. Clarke, that at a late period the matter is puriform, and the vesicle converted into a pustule, I am inclined to believe that it is erroneous; and that merely an inspissation of the fluid takes place, which may in some instances occasion it to resemble pus. The colour of the scab, which is totally different from that of dry pus, and its effects, justify this opinion.

I cannot therefore observe without regret, an hypothesis obstinately maintained, which, in consequence of the dangerous practice it inculcates, has already been productive of great mischief; and even endangered life. It is, indeed, so directly opposite to that of all other medical inoculators, tors, as far as I can recollect, that I for some time flattered myself, it might have been eradicated. This hope, however, has hitherto proved delusive; and others, as well as myself, have lately seen too convincing a proof, that it still prevails.

Having noticed this difference of opinion among the friends of vaccination, I shall now subjoin some intelligence lately received from Dr. De Carro; from which we may learn, in what estimation the opponents of the practice are held on the continent. He says, I am much obliged to you for your two publications. I knew Dr.———'s work; *it seems to be the produce either of old age, or of a crazy head.* However, if there are in England people stupid enough to be influenced by *such a dotard,* you have done right to shew *the state of his intellects.*

"Vaccination has no such opponents in Germany. For several years I have not seen or heard of any book against it; and know of no other opposition which it has to encounter, but the laziness and indifference of the people, particularly of the poor. The small-pox has again made its appearance at Vienna. We have had several exhortations on the subject from Government, and from medical practitioners; but I really think it is time to think of some means of coercion; which would perhaps cause some surprise at first, but soon be considered as legal as many other laws of medical police."

The following letters from Mr. Barker, transmitted by Dr. De Carro to Dr. Jenner, cannot fail to be considered as interesting by most of your readers.

*Extract of a Letter from* John Barker, *Esq. the British Consul at Aleppo, to* Wm. Hamilton, *Esq. Secretary to* Lord Elgin, *at Constantinople.*

The principal question, namely, whether the Arabian horses are subject to the *grease,* I can answer in the affirmative, from my own observation of those that I have kept. I am well informed, that it is also a very common disorder among the horses bred in the desert; where the small-pox makes dreadful ravages, and is always more or less prevalent. That the distemper called by us the *grease,* was probably introduced into England from this country, might be ascertained by discovering, whether it was known previous to the period that Arabian stallions were carried over, to improve the breed of running horses; a period, I should imagine, not very distant. Taking it for granted, that it was not known before this time, it may be said the Arabians ans treated us, as we treated the Americans with the small-pox; that they gave us the disease, without imparting the means of curing it, which the experience of ages had happily found out.

Soon after my establishment here, I was alarmed at the appearance of the grease, which I knew in England to be a serious malady, in one of my most valuable horses; but I was soon relieved from all anxiety about him, seeing with what facility my groom effected a complete cure. A short time afterwards, however, another was attacked, and as expeditiously relieved. A third succeeded, and a fourth; and, in short, perceiving that in the course of a few months, all my stud, consisting of eight horses, as well stallions as geldings of different ages, were attacked by the same distemper, I no longer entertained a doubt that it was infectious: and consequently resolved, if ever I should have another horse seized with it, to keep him separate from the rest.

I have since had only one or two infected with the grease; but I have had ample occasions to know, from my own experience, that it is a very common disorder in horses throughout Arabia; nay, it is the most common of any of the very few diseases to which the horses of this country are usually subject. The others are the cholic, the mange, and the farcy.

The grease attacks horses here chiefly in the depth of winter; and sometimes, but rarely, in the heat of summer. It may not be improper to mention likewise, that horses who have once had it are not thereby exempted from taking it a second time. On the contrary, those which have once been attacked, are observed to be the most susceptible of taking infection; so that it seems to have no other quality in common with the small-pox, but that of being contagious.

As to the question, whether persons who have the care of horses here are more or less liable to be attacked with the small-pox, it is impossible to resolve it, for this reason among many others, that such people have almost all had the small-pox before they became of an age to be fit to tend horses. Here follows the method of curing the grease; which consists principally of a poultice, made of Egyptian Henné (Lawsonia inermis Linn.) and Aloes Succotorin; pounded and mixed with vinegar.

*Letter of* Mr. Barker *to* Dr. De Carro, *dated Antioch, September* 1, 1805.

Although I have read with great attention your History of Vaccination in Turkey, &c. nothing has occurred to me worthy of remark on the subject of the grease in horses. The description of this disease, given by Dr. Sacco, is so conformable with my own observation, that I make no doubt it has in Italy precisely the same character, as it generally exhibits here in Arabian horses. However, I am inclined to believe, that the Arabian method of curing the grease, as described in my letter to Mr. Hamilton, is greatly preferable to the treatment mentioned by the Italian physician. By the early application of Henné and Aloes, I imagine also it will be found, that the suppuration will not come to a head, nor leave a deep scar.

It is remarkable, that although, in the first year of my residence at Aleppo, 1800, the grease went nearly through my whole stud, composed of eight or ten horses of different ages, and was also at the same time very prevalent in the stables of my acquaintances, I have since neither had a horse of my own attacked by that disease, nor has any other subject of the kind come under my observation. This would seem to shew, that the disorder is epidemic as well as contagious; and thus exhibits another property in common with the small-pox.

The next time I discover the grease in my stable, I will take care to keep an exact register of its progress, symptoms, &c. as far as may come within my knowledge; and do myself the pleasure of reporting to you the result of my observations.

Permit me, in the mean time, to remark a very trifling inaccuracy in the pamphlet which you have been good enough to send me. You speak incidentally, in your History of Vaccination in the East, of the Arabian manner of dressing their horses. Now, as I suppose you mean the Bedouin Arabs, or the inhabitants of the desert, by whom the best race of horses are bred, you must have been misinformed; because those people know not the use of any instrument for dressing or cleaning horses; that is, they never take any other care, than to fasten them to a picket by the leg or halter, and to give them their food; which, during the spring, consists of grass; and when the earth no longer produces that nutriment, they supply the want, not by barley or oats, but by camels milk; which is most assuredly preferable to any kind of grain.

Dr. De Carro thinks Mr. Barker's information corroborates.

ates the opinion, that the small-pox derives its origin from the grease; and that the matter may degenerate, and the disease become of a more malignant nature, from the total want of care and cleanliness, the growth of the hair, heat, and other causes. This is a point, which can only be determined by facts; and in the present instance, facts are not easily obtained. One circumstance, however, is here ascertained, which is, that the grease is not the mere consequence of domestication, as was hitherto supposed; but a common disorder among horses that are bred in the desert, and never housed. It appears, from the various accounts given of it, to be an erysipelatous eruption, commonly the effect of cold and moisture, and consequently, to be a chilblain. Hence it is most frequent in a cold and wet spring; and either unknown, or unfrequent, in the warm climate of Hindostan.

---

### *On the Danger of cutting up a Vaccine Vesicle.*

Having already stated, that I had known life endangered by cutting up a vaccine vesicle; and that the operation, serious as it is, was performed only for the sake of remedying an imaginary disease, I think it my duty here to state some particulars of that process, and of its effects; hoping that it may have a tendency to prevent a repetition of it in future.

The subject of it, Laura Watkins, No. 10, Leigh-street, Red Lion Square, was lately vaccinated in both arms; and her mother gives this account of the subsequent treatment of the case.

On the eighth day, when the areola was as large as a shilling, the inoculator pricked the vesicle on the right arm in several places, totally removed the cuticle from its surface, and wiped it out with the skirt of her frock. He then charged two lancets, three or four vaccinators, and a considerable number of glasses.

He caused the part to bleed, by the manner in which he took the matter on the vaccinators; and, when charging the glasses, he first drew the flat surfaces of them over the sore, and then scraped up more of the matter with their edges.—He also removed the cuticle from the vesicle on the left arm; but did not take any matter from it.

The patient was brought to him again on the tenth day, according to his direction. The inflammation was then as large as a crown piece. He then told Mrs. Watkins, her child was secure; and need not be brought to him any more.

 A few

A few days after, red spots appeared on the face, neck, forearms, and legs; and, in about twenty-four hours, a vesicle rose on the centre of each. These were succeeded by other eruptions, resembling a nettle-rash. From their being local, and entirely confined to those parts which are most exposed, there was reason to believe they were bug-bites; and that the vesicles which appeared on them for a few days, were occasioned by the increased irritability of the skin.

On the twentieth day, inflammation and tumefaction appeared on the right forearm; beginning at the elbow, and gradually advancing to the hand; and on the twenty-first, a swelling appeared in the axilla of the same side, attended with great pain. On this day an Apothecary was called in; who suspecting the stomach or bowels to be the seat of the complaint, directed a warm bath; and a dose of rhubarb and magnesia, which operated several times.

On the twenty-second day, the child was brought to me. She was reduced to a state of extreme debility. The inflammation and tumefaction were then principally confined to the wrist and hand. The axillary glands were enlarged; and her arm could not be moved without occasioning great pain. I directed, that she should be kept in a recumbent posture, with the hand and arm elevated; and that a compress with cold water should be frequently applied to the part affected. By these means the symptoms gradually disappeared; first at the wrist, and afterwards at the hand; the interstitial fluid being gradually absorbed, and the inflammatory diathesis subdued; but the hand continued rather œdematous for some days.

A diarrhœa occurred soon after, which once more threatened danger; but yielded to the medicines which were administered; and she is now likely to recover her health. This case was seen by Dr. Jenner, and several other medical men; all of whom testified their astonishment at the repetition of such a rash experiment, after so many admonitions; and at such an obstinate perseverance in error.

If any thing can exceed the surprise occasioned by this recent instance of mal-practice in spite of every warning, it is the confidence with which some persons attempt, at one time to palliate such proceedings, at another to justify, and at another to deny them. It is also a little remarkable, that these glaring instances of misconduct have prevailed most in that quarter, where it was most natural to expect an example of good practice, and a rigid compliance ance with rational instructions. That such instructions were given, and given by those who had authority to give them, is well known; although the contrary has been more than once asserted.

Among the instructions here alluded to, are the following; which, unfortunately, it is still necessary to repeat.

1. " An accurate knowledge of the signs of infection, and of *the character and progress of the vaccine vesicle*, is essential to the success of this inoculation.

2. " The success of the operation is doubtful, where there is any considerable deviation from *the usual course of the disease*.

3. " Matter is to be taken by small superficial punctures, made in several parts of the vesicle, with a lancet introduced horizontally.—*Time should be allowed for the fluid to exude;* which will appear on the vesicle, in the form of small pellucid drops.—If necessary, *very slight pressure* may be applied with the flat surface of the lancet, to quicken the discharge.

4. " *Vaccinators require much less matter to charge them, than thread or glass.* When they are not intended to be used soon, they ought to be repeatedly charged. Every practitioner who has not a constant succession of patients, ought to take matter when he has an opportunity; and preserve it for any future occasion.

5. " *An assurance of security, from vaccine inoculation, can only be obtained by carefully observing the whole progress of the disease. If any doubt remain, the operation ought to be repeated.*

Among other circumstances, which the inoculator is enjoined to notice, by the instructions in question, are the peculiar areola with which the vesicle is surrounded, and the peculiar scab into which it is converted; circumstances which it is impossible to notice when the vesicle is destroyed at an early period, and never re-appears.

According to the foregoing regulations, which were sanctioned by no mean authority, it is necessary to be cautious in taking matter, and economical in its distribution. It is also necessary to watch the whole progress of vaccination; and if any material deviation from the usual course of the process occurs, to inoculate the patient again.

---

I some time ago received from Mr. Key, surgeon, in Fenchurch Street, a statement of cases, in which vaccina-

 tion tion was supposed to have failed. They did not occur in his own practice; and the documents transmitted with them did not appear to me sufficient to warrant a conclusion, that the failures really occurred.

The intention of the communication was, to bring forward evidence, in addition to what has been already advanced by some authors, that vaccination is only a temporary protection against the small-pox; which these cases seemed to indicate, because the small-pox was more violent in that case, in which the longest time had elapsed since vaccination.

I have made very extensive inquiries into this subject; and am more and more confirmed in opinion, that no conclusion of the kind is warranted by the cases of failure which have occurred; and that no gradual diminution of the security against the small-pox, arising from the cow-pock, takes place.

On the present occasion, I need not produce any stronger instances to the contrary, than those in Fullwood's Rents, in which the child who had been vaccinated the longest period had the small-pox by far most severely. To this observation may be added that of Dr. Willan, in his Treatise on Vaccine Inoculation; who expresses himself as follows.

"I will not offer the arguments from analogy, which have been employed by several writers, in answer to the opinion that vaccine inoculation is only a temporary preventive of the small-pox. The supposition does not rest upon either probable or consistent grounds; as the cases of variolous eruption, adduced above, took place without any certain order, from five months to seven years after vaccination. If it be said, that the preventive power of the cow-pock ceases in some persons at the end of a month or two, while in others it lasts sixty or seventy years, according to the varieties of constitution, the assertion is too vague to admit of an answer. Most of the persons mentioned were vaccinated in 1800. This does not prove that the effect of vaccination is removed in three or four years; but rather confirms a preceding remark, that the circumstances requisite to produce the full effect, were not ascertained in the first year or two of the new practice.

"Some gentlemen with whom I have conversed, maintain that the symptoms enumerated, page 69,—70, occur more or less extensively in all cases, according to the time between vaccination and the application of variolous matter; ter; and that at length the constitution would regain the susceptibility of the small-pox; the preventive power of vaccine inoculation having been gradually exhausted. I ascertained the incorrectness of this opinion, while I superintended the inoculations to which I referred, page 15; but I am happy to cite on the subject, the authority of the physicians and surgeons of the Inoculation Hospital, and of the Vaccine Pock Institution, who have uniformly found, that the degree of fever, inflammation, or eruption, in the few cases where they occur, were "not according to the length of time after vaccination," but depending, in some persons, on the state of the constitution, and in others, on incidental circumstances. "The appearances of the inoculated part depend on the two diluted state of the fluid, or its altered state by keeping; on the kind of wound made in inoculation; on external injury, or irritation from pressure of clothes, scratching, &c. and on the constitution of the patient.

"Mr. Goldson seems to think, that some eruptive, or contagious diseases, such as the measles, the scarlatina, the chicken-pox, the nettle-rash, the hooping-cough, and the dysentery, may, by altering the state of the skin, remove the security derived from the cow-pock.—I have found on inquiry, that more than half the children above mentioned, who took the small-pox after vaccination, had been intermediately affected with the measles, &c. but that in the rest there had not been any intervening disease."

Mr. Goldson seems determined, at all events, to maintain his opinion, that vaccination is only a very fallacious and precarious kind of security against the small-pox; and that its effect wears out. Though foiled again and again, he still shews *bottom,* and returns to the charge. I trust, however, that the preceding quotations, and the respectable authorities referred to, are amply sufficient to refute such an idle and unsupported hypothesis; and set this question to rest.

I am, &c.

JOHN RING.

*New Street, Hanover Square,*
*September* 12, 1806.

